# Ethyl 2-(1*H*-1,2,3-benzotriazol-1-yl)acetate

**DOI:** 10.1107/S160053681005138X

**Published:** 2010-12-15

**Authors:** Xiao-Xia Li, Zhong Chen

**Affiliations:** a Institute of Functional Materials, Jiangxi University of Finance & Economics, Nanchang 330013, People’s Republic of China

## Abstract

The title compound, C_10_H_11_N_3_O_2_, was synthesized by the reaction of 1*H*-benzotriazole with ethyl 2-chloro­acetate in ethanol. The non-H atoms, excluding the benzotriazol-1-yl group, are almost coplanar (r.m.s. deviation of the non-H atoms = 0.0409 Å). The dihedral angle formed between this plane and the benzotriazole ring is 79.12 (5)° In the crystal, weak inter­molecular C—H⋯N and C—H⋯O inter­actions help to consolidate the three-dimensional network.

## Related literature

For related structures, see: Shi *et al.* (2007*a* ▶,*b* ▶); Ji *et al.* (2008 ▶); Zhang *et al.* (2009 ▶).
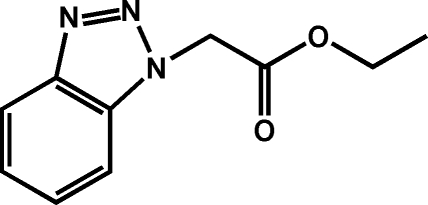


## Experimental

### 

#### Crystal data

C_10_H_11_N_3_O_2_
*M* *_r_* = 205.22
Monoclinic, 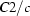

*a* = 20.6734 (9) Å
*b* = 11.9284 (5) Å
*c* = 9.3420 (4) Å
β = 111.770 (3)°
*V* = 2139.44 (16) Å^3^
*Z* = 8
Mo *K*α radiation
μ = 0.09 mm^−1^
*T* = 296 K
0.23 × 0.18 × 0.16 mm

#### Data collection

Bruker SMART APEX CCD diffractometer
Absorption correction: multi-scan (*SADABS*; Bruker, 2007 ▶) *T*_min_ = 0.980, *T*_max_ = 0.985
9605 measured reflections
2551 independent reflections
1360 reflections with *I* > 2σ(*I*)
*R*_int_ = 0.039

#### Refinement

*R*[*F* ^2^ > 2σ(*F* ^2^)] = 0.067
*wR*(*F* ^2^) = 0.231
*S* = 1.07
2551 reflections
137 parameters
1 restraint
H-atom parameters constrained
Δρ_max_ = 0.50 e Å^−3^
Δρ_min_ = −0.24 e Å^−3^

### 

Data collection: *APEX2* (Bruker, 2007 ▶); cell refinement: *SAINT* (Bruker, 2007 ▶); data reduction: *SAINT*; program(s) used to solve structure: *SHELXS97* (Sheldrick, 2008 ▶); program(s) used to refine structure: *SHELXL97* (Sheldrick, 2008 ▶); molecular graphics: *SHELXTL* (Sheldrick, 2008 ▶); software used to prepare material for publication: *SHELXTL*.

## Supplementary Material

Crystal structure: contains datablocks I, global. DOI: 10.1107/S160053681005138X/vm2062sup1.cif

Structure factors: contains datablocks I. DOI: 10.1107/S160053681005138X/vm2062Isup2.hkl

Additional supplementary materials:  crystallographic information; 3D view; checkCIF report

## Figures and Tables

**Table 1:** Hydrogen-bond geometry (Å, °)

*D*—H⋯*A*	*D*—H	H⋯*A*	*D*⋯*A*	*D*—H⋯*A*
C2—H2*A*⋯N2^i^	0.93	2.57	3.457 (3)	159
C7—H7*A*⋯N3^i^	0.97	2.51	3.387 (3)	150
C7—H7*B*⋯O1^ii^	0.97	2.49	3.451 (3)	173

Symmetry codes: (i) 
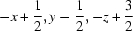
; (ii) 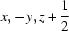.

**Fig. 1.**
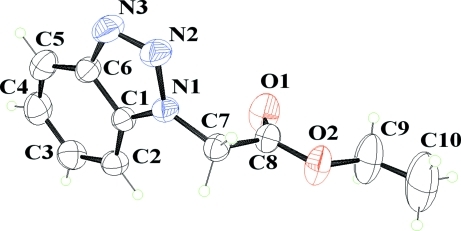
The title molecule with the displacement ellipsoids shown at the 30% probability level.

**Fig. 2.**
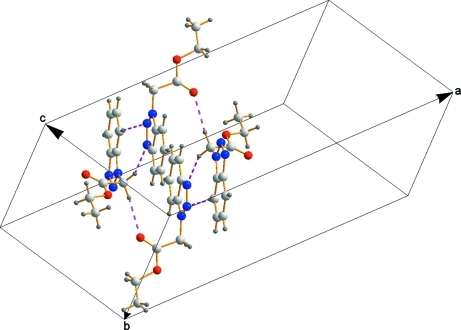
The crystal packing for the title compound via C—H···O and C—H···N weak interactions shown as dashed lines.

## supplementary crystallographic information

### Comment

The Schiff-bases derived from benzotriazol-1-yl-acetic acid hydrazide with a relevant aldehyde or ketone have been widely investigated in recent years (Shi *et al.*, 2007*a*; Shi *et al.*, 2007*b*; Ji *et al.*, 2008). As an important intermediate in the synthesis procedure of benzotriazol-1-yl-acetic acid hydrazide (Zhang *et al.*, 2009), the title compound, (I), was synthesized and characterized by X-ray diffraction. The asymmetric unit of (I) comprises one independent molecule (Fig. 1). All the bond lengths are comparable with those observed in benzotriazol-1-yl-acetic acid hydrazide. In the crystal, weak intermolecular C—H···N and C—H···O interactions are helpful to consolidate the three-dimensional network (Fig. 2, Table 1).

### Experimental

1*H*-benzotriazole (1 mmol) and sodium hydroxide (1 mmol) were dissolved in ethanol (10 ml), and an ethanol (5 ml) solution of ethyl 2-chloroacetate (1 mmol) was added dropwise. After stirring for 4 h at room temperature, the mixture was filtered and set aside to crystallize at room temperature for several days, giving colourless block crystals.

### Refinement

The C7—C8 bond length was restrainted to a reasonable distance: C7—C8 = 1.501 (4) Å (command *DFIX*). All H atoms were situated at idealized positions with the carrier atom-H distances = 0.93 Å for aryl, 0.97 for methylene, 0.96 Å for the methyl. The *U*_iso_ values were constrained to be 1.5*U*_eq_ of the carrier atom for the methyl H atoms and 1.2*U*_eq_ for the remaining H.

### Crystal data

**Table:**

C_10_H_11_N_3_O_2_	*F*(000) = 864
*M**_r_* = 205.22	*D*_x_ = 1.274 Mg m^−^^3^
Monoclinic, *C*2/*c*	Mo *K*α radiation, λ = 0.71073 Å
Hall symbol: -C 2yc	Cell parameters from 1764 reflections
*a* = 20.6734 (9) Å	θ = 2.8–21.8°
*b* = 11.9284 (5) Å	µ = 0.09 mm^−^^1^
*c* = 9.3420 (4) Å	*T* = 296 K
β = 111.770 (3)°	Block, colourless
*V* = 2139.44 (16) Å^3^	0.23 × 0.18 × 0.16 mm
*Z* = 8	

### Data collection

**Table:**

Bruker SMART APEX CCD diffractometer	2551 independent reflections
Radiation source: fine-focus sealed tube	1360 reflections with *I* > 2σ(*I*)
graphite	*R*_int_ = 0.039
φ and ω scans	θ_max_ = 27.9°, θ_min_ = 2.0°
Absorption correction: multi-scan (*SADABS*; Bruker, 2007)	*h* = −24→27
*T*_min_ = 0.980, *T*_max_ = 0.985	*k* = −15→13
9605 measured reflections	*l* = −12→12

### Refinement

**Table:**

Refinement on *F*^2^	Secondary atom site location: difference Fourier map
Least-squares matrix: full	Hydrogen site location: inferred from neighbouring sites
*R*[*F*^2^ > 2σ(*F*^2^)] = 0.067	H-atom parameters constrained
*wR*(*F*^2^) = 0.231	*w* = 1/[σ^2^(*F*_o_^2^) + (0.1115*P*)^2^ + 0.7271*P*] where *P* = (*F*_o_^2^ + 2*F*_c_^2^)/3
*S* = 1.07	(Δ/σ)_max_ < 0.001
2551 reflections	Δρ_max_ = 0.50 e Å^−^^3^
137 parameters	Δρ_min_ = −0.24 e Å^−^^3^
1 restraint	Extinction correction: *SHELXL97* (Sheldrick, 2008), Fc^*^=kFc[1+0.001xFc^2^λ^3^/sin(2θ)]^-1/4^
Primary atom site location: structure-invariant direct methods	Extinction coefficient: 0.0044 (15)

### Special details

**Table:**

Geometry. All e.s.d.'s (except the e.s.d. in the dihedral angle between two l.s. planes) are estimated using the full covariance matrix. The cell e.s.d.'s are taken into account individually in the estimation of e.s.d.'s in distances, angles and torsion angles; correlations between e.s.d.'s in cell parameters are only used when they are defined by crystal symmetry. An approximate (isotropic) treatment of cell e.s.d.'s is used for estimating e.s.d.'s involving l.s. planes.
Refinement. Refinement of *F*^2^ against ALL reflections. The weighted *R*-factor *wR* and goodness of fit *S* are based on *F*^2^, conventional *R*-factors *R* are based on *F*, with *F* set to zero for negative *F*^2^. The threshold expression of *F*^2^ > σ(*F*^2^) is used only for calculating *R*-factors(gt) *etc*. and is not relevant to the choice of reflections for refinement. *R*-factors based on *F*^2^ are statistically about twice as large as those based on *F*, and *R*- factors based on ALL data will be even larger.

### Fractional atomic coordinates and isotropic or equivalent isotropic displacement parameters (Å^2^)

**Table:**

	*x*	*y*	*z*	*U*_iso_*/*U*_eq_	
C1	0.22410 (13)	−0.01039 (19)	0.6218 (3)	0.0558 (6)	
C2	0.19757 (14)	−0.1116 (2)	0.5489 (3)	0.0682 (7)	
H2A	0.2148	−0.1805	0.5934	0.082*	
C3	0.14480 (17)	−0.1032 (3)	0.4084 (3)	0.0850 (9)	
H3A	0.1254	−0.1685	0.3554	0.102*	
C4	0.11878 (19)	0.0009 (3)	0.3412 (4)	0.0953 (11)	
H4A	0.0829	0.0026	0.2448	0.114*	
C5	0.14479 (17)	0.0994 (3)	0.4138 (3)	0.0847 (9)	
H5A	0.1273	0.1683	0.3695	0.102*	
C6	0.19825 (14)	0.0923 (2)	0.5557 (3)	0.0646 (7)	
C7	0.31980 (13)	−0.0550 (2)	0.8782 (3)	0.0599 (7)	
H7A	0.2938	−0.1200	0.8893	0.072*	
H7B	0.3356	−0.0143	0.9751	0.072*	
C8	0.38177 (14)	−0.0931 (2)	0.8442 (3)	0.0656 (7)	
C9	0.4915 (2)	−0.1815 (5)	0.9535 (4)	0.1336 (18)	
H9A	0.4811	−0.2436	0.8814	0.160*	
H9B	0.5121	−0.1219	0.9141	0.160*	
C10	0.5370 (3)	−0.2152 (7)	1.0919 (6)	0.193 (3)	
H10A	0.5790	−0.2408	1.0814	0.289*	
H10B	0.5168	−0.2753	1.1299	0.289*	
H10C	0.5474	−0.1536	1.1630	0.289*	
N1	0.27471 (10)	0.01616 (16)	0.7579 (2)	0.0587 (6)	
N2	0.28073 (12)	0.12909 (18)	0.7742 (3)	0.0746 (7)	
N3	0.23544 (14)	0.17555 (18)	0.6542 (3)	0.0791 (7)	
O1	0.39013 (12)	−0.0827 (2)	0.7269 (2)	0.1030 (8)	
O2	0.42674 (10)	−0.1412 (2)	0.9665 (2)	0.0926 (8)	

### Atomic displacement parameters (Å^2^)

**Table:**

	*U*^11^	*U*^22^	*U*^33^	*U*^12^	*U*^13^	*U*^23^
C1	0.0550 (14)	0.0542 (14)	0.0614 (14)	0.0012 (11)	0.0252 (11)	0.0022 (11)
C2	0.0740 (18)	0.0594 (15)	0.0700 (16)	−0.0027 (13)	0.0252 (14)	−0.0003 (12)
C3	0.086 (2)	0.088 (2)	0.0721 (18)	−0.0117 (17)	0.0185 (16)	−0.0098 (16)
C4	0.079 (2)	0.122 (3)	0.0710 (19)	0.005 (2)	0.0117 (16)	0.0104 (19)
C5	0.086 (2)	0.085 (2)	0.0772 (19)	0.0188 (17)	0.0246 (17)	0.0170 (16)
C6	0.0655 (16)	0.0586 (15)	0.0730 (16)	0.0084 (12)	0.0295 (14)	0.0083 (12)
C7	0.0588 (15)	0.0603 (15)	0.0571 (13)	0.0023 (11)	0.0173 (11)	0.0007 (11)
C8	0.0630 (16)	0.0714 (17)	0.0584 (15)	0.0066 (13)	0.0179 (12)	0.0009 (12)
C9	0.088 (3)	0.212 (5)	0.099 (3)	0.066 (3)	0.032 (2)	0.013 (3)
C10	0.095 (3)	0.328 (8)	0.153 (4)	0.078 (4)	0.043 (3)	0.042 (5)
N1	0.0619 (13)	0.0470 (11)	0.0653 (12)	0.0005 (9)	0.0214 (10)	−0.0025 (9)
N2	0.0837 (16)	0.0490 (12)	0.0866 (16)	0.0008 (11)	0.0264 (13)	−0.0048 (11)
N3	0.0919 (18)	0.0531 (13)	0.0878 (16)	0.0087 (12)	0.0282 (14)	0.0035 (12)
O1	0.0971 (17)	0.151 (2)	0.0722 (13)	0.0321 (14)	0.0445 (12)	0.0153 (13)
O2	0.0721 (13)	0.1303 (19)	0.0729 (12)	0.0370 (13)	0.0240 (10)	0.0187 (12)

### Geometric parameters (Å, °)

**Table:**

C1—N1	1.351 (3)	C7—H7A	0.9700
C1—C6	1.387 (3)	C7—H7B	0.9700
C1—C2	1.395 (3)	C8—O1	1.177 (3)
C2—C3	1.364 (4)	C8—O2	1.309 (3)
C2—H2A	0.9300	C9—C10	1.348 (5)
C3—C4	1.405 (5)	C9—O2	1.468 (4)
C3—H3A	0.9300	C9—H9A	0.9700
C4—C5	1.364 (5)	C9—H9B	0.9700
C4—H4A	0.9300	C10—H10A	0.9600
C5—C6	1.378 (4)	C10—H10B	0.9600
C5—H5A	0.9300	C10—H10C	0.9600
C6—N3	1.378 (3)	N1—N2	1.356 (3)
C7—N1	1.441 (3)	N2—N3	1.289 (3)
C7—C8	1.501 (4)		
			
N1—C1—C6	104.3 (2)	H7A—C7—H7B	107.9
N1—C1—C2	133.6 (2)	O1—C8—O2	123.9 (3)
C6—C1—C2	122.1 (2)	O1—C8—C7	126.7 (3)
C3—C2—C1	115.8 (3)	O2—C8—C7	109.5 (2)
C3—C2—H2A	122.1	C10—C9—O2	110.6 (3)
C1—C2—H2A	122.1	C10—C9—H9A	109.5
C2—C3—C4	122.1 (3)	O2—C9—H9A	109.5
C2—C3—H3A	118.9	C10—C9—H9B	109.5
C4—C3—H3A	118.9	O2—C9—H9B	109.5
C5—C4—C3	121.6 (3)	H9A—C9—H9B	108.1
C5—C4—H4A	119.2	C9—C10—H10A	109.5
C3—C4—H4A	119.2	C9—C10—H10B	109.5
C4—C5—C6	116.9 (3)	H10A—C10—H10B	109.5
C4—C5—H5A	121.5	C9—C10—H10C	109.5
C6—C5—H5A	121.5	H10A—C10—H10C	109.5
N3—C6—C5	130.4 (3)	H10B—C10—H10C	109.5
N3—C6—C1	108.2 (2)	C1—N1—N2	110.22 (19)
C5—C6—C1	121.4 (3)	C1—N1—C7	130.4 (2)
N1—C7—C8	111.7 (2)	N2—N1—C7	119.4 (2)
N1—C7—H7A	109.3	N3—N2—N1	108.8 (2)
C8—C7—H7A	109.3	N2—N3—C6	108.5 (2)
N1—C7—H7B	109.3	C8—O2—C9	116.4 (2)
C8—C7—H7B	109.3		
			
N1—C1—C2—C3	179.7 (3)	C2—C1—N1—N2	179.5 (3)
C6—C1—C2—C3	0.3 (4)	C6—C1—N1—C7	179.3 (2)
C1—C2—C3—C4	0.0 (5)	C2—C1—N1—C7	−0.2 (5)
C2—C3—C4—C5	−0.4 (5)	C8—C7—N1—C1	83.0 (3)
C3—C4—C5—C6	0.5 (5)	C8—C7—N1—N2	−96.6 (3)
C4—C5—C6—N3	178.7 (3)	C1—N1—N2—N3	0.6 (3)
C4—C5—C6—C1	−0.3 (4)	C7—N1—N2—N3	−179.7 (2)
N1—C1—C6—N3	1.1 (3)	N1—N2—N3—C6	0.1 (3)
C2—C1—C6—N3	−179.3 (2)	C5—C6—N3—N2	−179.9 (3)
N1—C1—C6—C5	−179.7 (2)	C1—C6—N3—N2	−0.8 (3)
C2—C1—C6—C5	−0.1 (4)	O1—C8—O2—C9	1.3 (5)
N1—C7—C8—O1	−10.6 (4)	C7—C8—O2—C9	−178.8 (3)
N1—C7—C8—O2	169.5 (2)	C10—C9—O2—C8	171.7 (5)
C6—C1—N1—N2	−1.0 (3)		

### Hydrogen-bond geometry (Å, °)

**Table:**

*D*—H···*A*	*D*—H	H···*A*	*D*···*A*	*D*—H···*A*
C2—H2A···N2^i^	0.93	2.57	3.457 (3)	159
C7—H7A···N3^i^	0.97	2.51	3.387 (3)	150
C7—H7B···O1^ii^	0.97	2.49	3.451 (3)	173

Symmetry codes: (i) −*x*+1/2, *y*−1/2, −*z*+3/2; (ii) *x*, −*y*, *z*+1/2.
